# Supplementary figures and images for: American crocodiles (Crocodylus acutus) as restoration bioindicators in the Florida Everglades
Source: PLoS One. 2021 May 19;16(5):e0250510. doi: 10.1371/journal.pone.0250510 (PMC8133456; doi:10.1371/journal.pone.0250510)

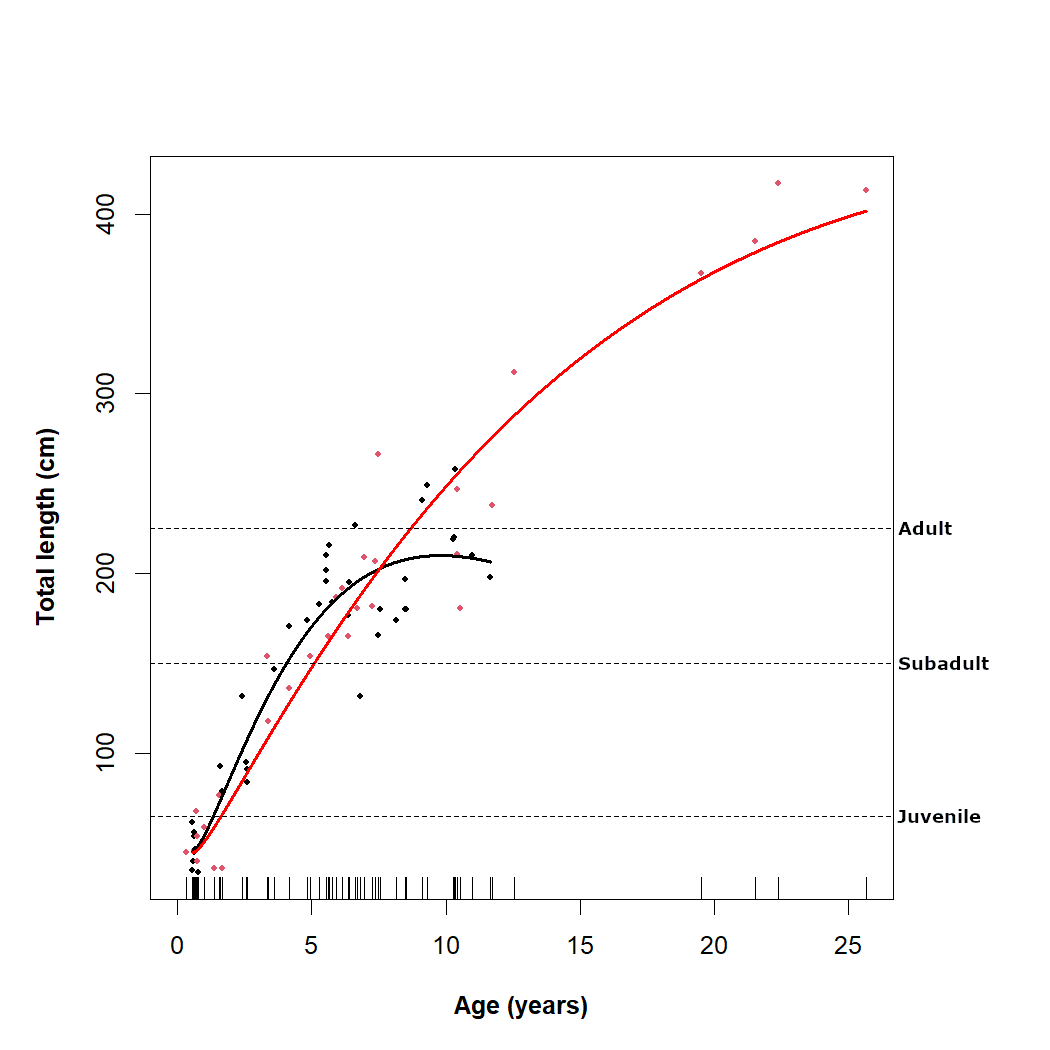

Supplement: S1 Fig — Males are represented as red circles with a Loess best-fit line in red and females are represented as black circles with a Loess best-fit line in black. Horizontal dashed lines represent size classes. (TIF) [file pone.0250510.s001.tif]

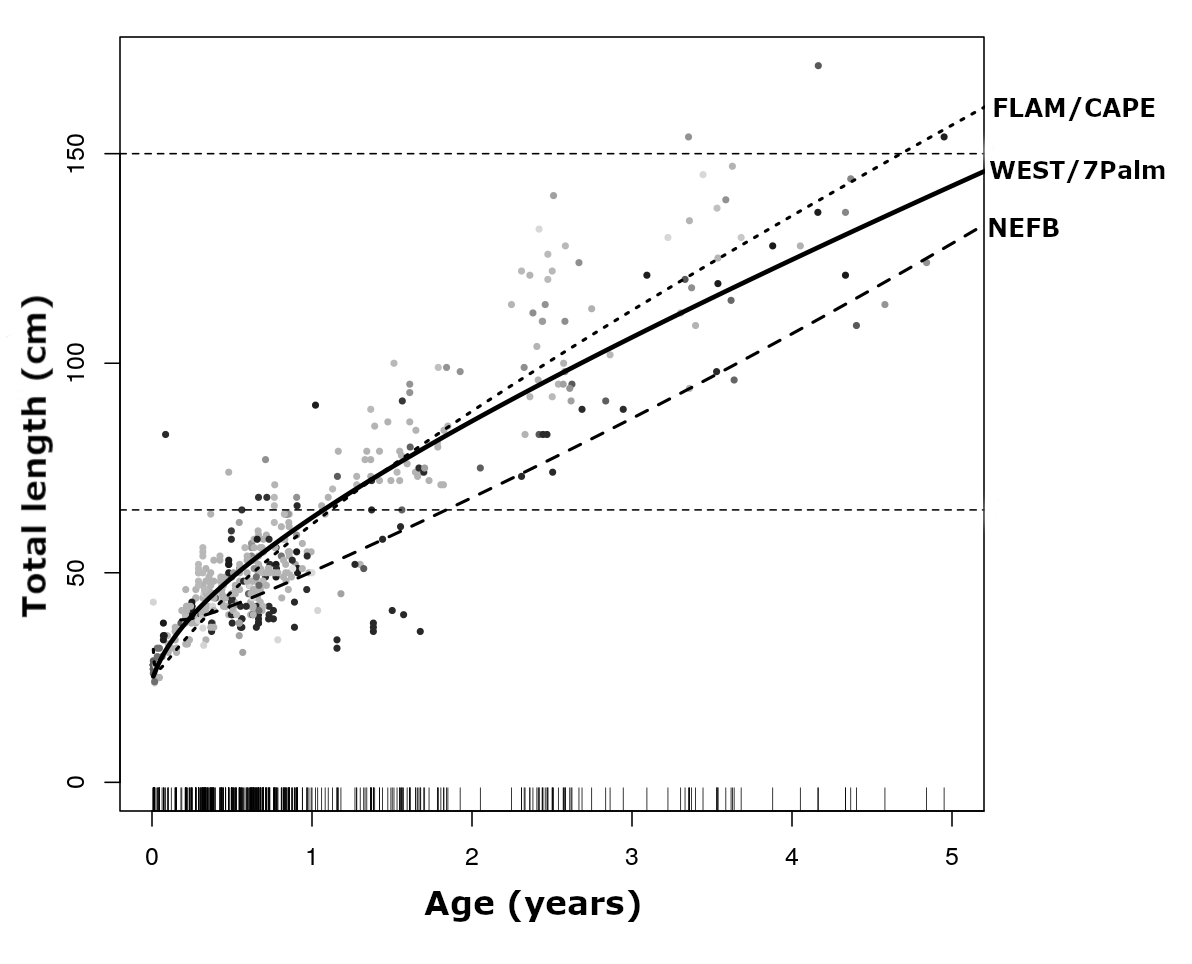

Supplement: S2 Fig — Solid line represents average growth in West Lake and Seven Palm (7Palm) areas, dotted line is average growth rate at Flamingo and Cape Sable areas, and dashed line is average growth rates in NE Florida Bay. Horizontal dashed lines represent size classes within first five years: Juvenile and subadult. (TIF) [file pone.0250510.s002.tif]

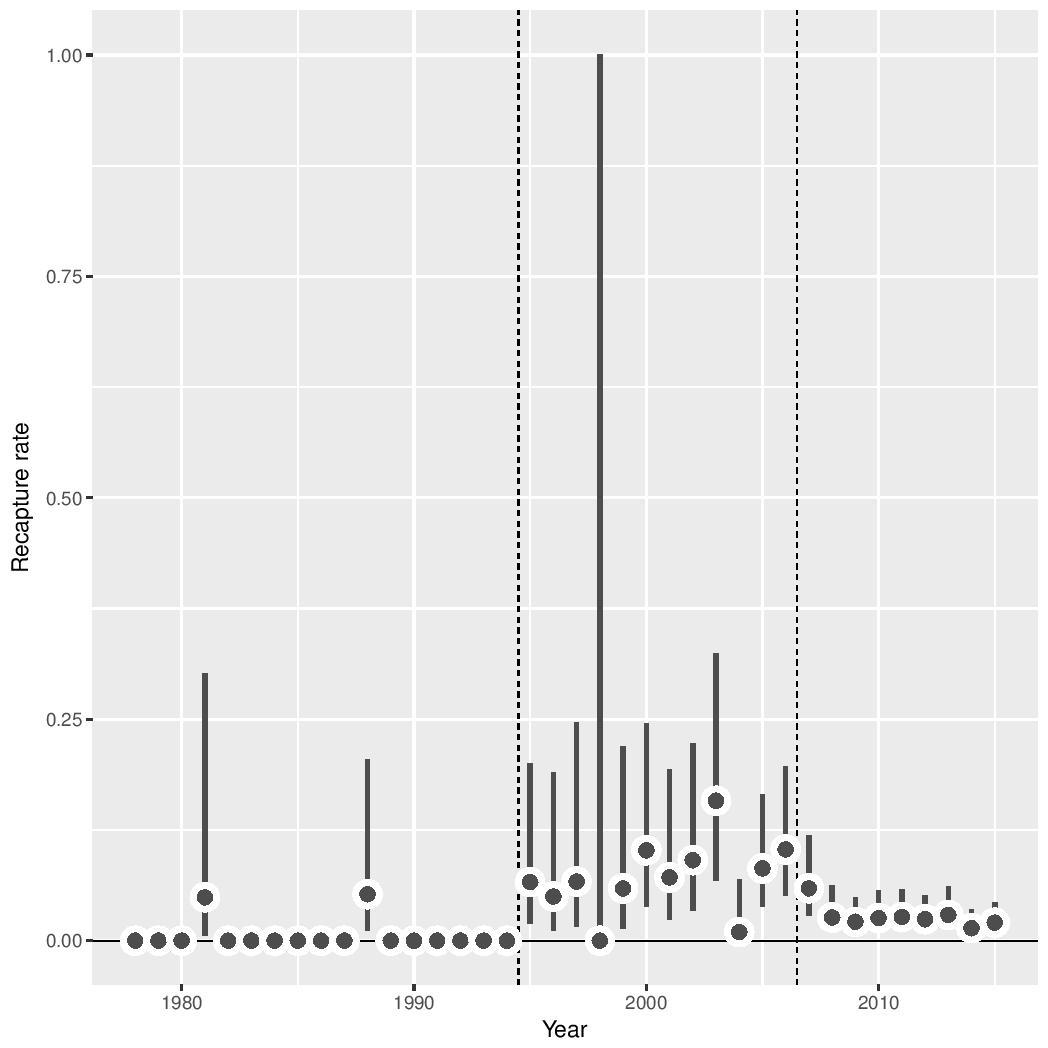

Supplement: S3 Fig — Dots represent mean values and lines indicate 95% confidence intervals, dashed lines represent different phases of similar recapture rates (before 1995, 1995–2006, 2007–2015). (TIF) [file pone.0250510.s003.tif]

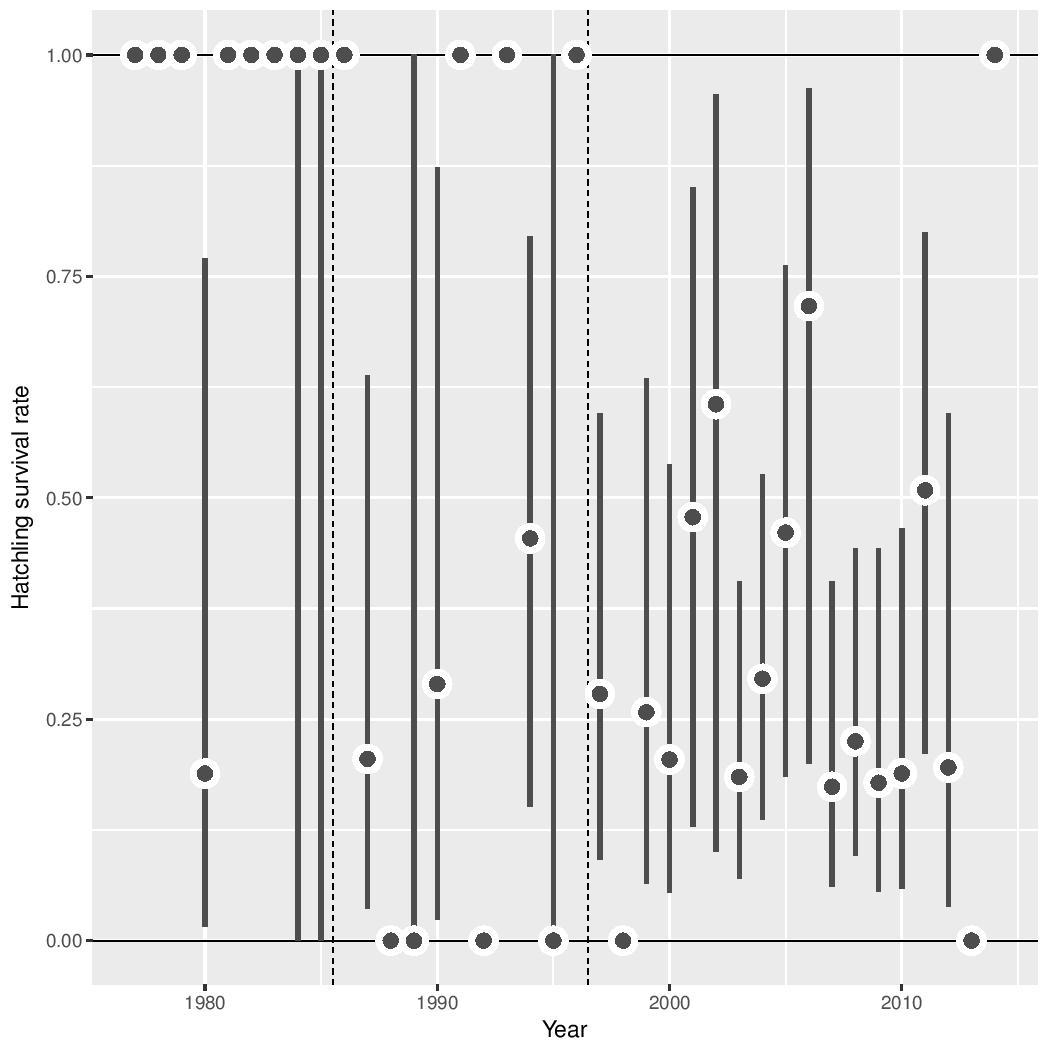

Supplement: S4 Fig — Dots represent mean values and error bars are 95% confidence intervals, dashed lines reflect phases of recapture rate. (TIF) [file pone.0250510.s004.tif]
