# Supplementary material for: American crocodiles (Crocodylus acutus) as restoration bioindicators in the Florida Everglades
Source: PLoS One. 2021 May 19;16(5):e0250510. doi: 10.1371/journal.pone.0250510 (PMC8133456; doi:10.1371/journal.pone.0250510)
Supplement: S1 Table — (DOCX) [file pone.0250510.s005.docx]

**S1 Table. Timeline of Restoration Events in the Florida Everglades**

LEGISLATIVE ACTION

1989 Modified Water Deliveries to Everglades National Park (ENP) *First legislation targeting ecosystem restoration*

1992 Kissimmee River Restoration Project

1996 Water Resources Development Act (WRDNA) - Critical Projects Authorized C-111 South Dade Project Authorized

1999 Central & Southern Florida Comprehensive Review Study (Yellow Book)

2000 WRDA - Comprehensive Everglades Restoration Plan (CERP) Authorized

2007 WRDA - Generation 1 CERP Projects Authorized

2014 WRRDA - Generation 2 CERP Projects Authorized

2016 Water Infrastructure Improvements for the Nation (WIIN) Act - Central Everglades Planning Project (CEPP) Authorized

2050 Completion of CERP scheduled

**PROJECT IMPLEMENTATION**

1956 Plug put in at Cape Sable to reduce saltwater intrusion

1969–1980 Water supplied to Taylor River via Taylor Slough

1981–1999 Taylor Slough pump installed and functioning

1986 Canals plugged by National Park Service

1992 East Cape Canal Extension and Homestead Canal dams fail

1997 Dams replaced at East Cape Canal and Homestead Canal

2005–2007 Cape Sable plug failed

2007 New plugs constructed; new dams constructed at Cape Sable

2010–2011 Dams replaced at East Cape Canal and Homestead Canal

2011 C-111 Spreader canal constructed; some flow restored to Florida Bay

2011 Canals plugged by NPS

2015 Water supplied to Shark Slough (north of Taylor Slough) with a small increase in flow

2016 Site 1 Impoundment reduce seepage loss from Water Conservation Area-1 (WCA-1), recharge groundwater supply

2017 Seminole Big Cypress – restore wetlands in Big Cypress Basin Reservation

2017 C-51 Stormwater Treatment area - stormwater treatment from C-51 Canal discharge into WCA-1

2018 Modified Water Deliveries (ENP) - Improve natural water flow to ENP, residential flood control, reconnect freshwater flows

2018 C-111 South Dade reduce quantity of water discharged into Barnes sound and seepage out of ENP

2020 Kissimmee River Restoration – restore timing and distribution of flow into Lake Okeechobee

_____________________________________________________________________________________

Everglades restoration will enable the right quantity of water, at the right quality, to be distributed to the right place, at the right time throughout South Florida. This will be accomplished through the implementation of multiple projects that will work together to provide: Water Storage, Water Treatment, Water Conveyance, and Water Distribution (USACE 2018).
